# Narrowing Down Key Players in Autoimmunity via Single‐Cell Multiomics

**DOI:** 10.1002/eji.202451233

**Published:** 2025-06-19

**Authors:** Altea Gjurgjaj, Cecilia Domínguez Conde

**Affiliations:** ^1^ Human Technopole Milan Italy

**Keywords:** antigen specificity, autoimmunity, cell states, molecular mimicry, single‐cell sequencing

## Abstract

Autoimmune diseases encompass a range of conditions in which our own immune system reacts against molecules encoded by our own genome. This phenomenon is mediated by the action of antigen receptors expressed by T and B cells. Identifying the molecular events that trigger these responses as well as the effector cells that underlie them is at the heart of autoimmunity research. In this review, we discuss how single‐cell multiomics techniques applied to healthy and patient tissues are shedding light on the mechanisms underpinning autoimmune conditions, specifically by identifying disease‐associated cell states and cellular communication networks, including those linked to specific autoimmunity susceptibility genetic loci. Furthermore, we dive into the unprecedented resolution achieved in mapping autoreactive lymphocytes, a key component of autoimmune responses. We conclude with a perspective on key bottlenecks and promising future directions leveraging the latest advances in single‐cell sequencing with orthogonal methods.

## Graphical abstract

This review explores how single‐cell sequencing and multiomics reveal cell states and communication networks involved in autoimmunity. We highlight insights into antigen specificity and autoreactive lymphocytes, uncovering mechanisms like molecular mimicry. We discuss genetic susceptibility loci in healthy and disease tissues and the challenges and opportunities in integrating different approaches to better understand autoimmune disease.

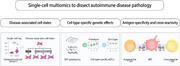

## 1 Introduction

The immune system is a sophisticated biological network of cells with highly specialized functions. In homeostatic conditions, these cells maintain a balance between effectively mounting immune responses against harmful pathogens or malignant cells and preventing autoimmune responses against self‐antigens. However, aberrant activation of antigen‐specific responses, mediated by B and T cells, can result in the recognition and destruction of healthy tissues leading to a broad landscape of autoimmune diseases that affect an estimated 10% of the global population [1, 2, 3].

Autoimmunity research strives to dissect the mechanisms causing autoimmune diseases to design more effective and targeted therapies. The causes are multifactorial, including genetic and environmental factors. Despite the heterogeneity across autoimmune diseases, many patients receive disease‐agnostic symptomatic treatments that do not tackle the disease‐triggering events [4]. The lack of disease‐specific curative treatments highlights the need for improving our understanding of the underlying pathogenesis of autoimmune disorders and, consequently, refine our therapeutic strategies.

Recent advances in multimodal single‐cell sequencing technologies offer the opportunity to identify disease‐associated cell subsets in human tissues at an unprecedented breadth and resolution [5]. In healthy individuals, collections of cellular profiles are being integrated into cell atlases, providing a comprehensive view of cell types and states across tissues [6]. In recent years, these approaches have also played a vital role in gaining deeper insights into cell states that arise in pathological conditions including autoimmunity. In this review, we discuss how single‐cell approaches have recently contributed to pushing forward autoimmunity research and we point out potential future directions in this field. First, we highlight some of the major efforts leveraging single‐cell RNA sequencing (scRNAseq) to narrow down specific cellular phenotypes enriched or restricted to autoimmune patient tissues, including specific subtypes of fibroblasts and T cells. Second, we summarize the major efforts linking genetic associations of autoimmune conditions to single‐cell expression data. Lastly, we discuss recent studies focused on the deep characterization of antigen‐specific responses.

## 2 Identifying Stromal and Immune Cell States in Autoimmunity with Single‐Cell Technologies

**FIGURE 1:**
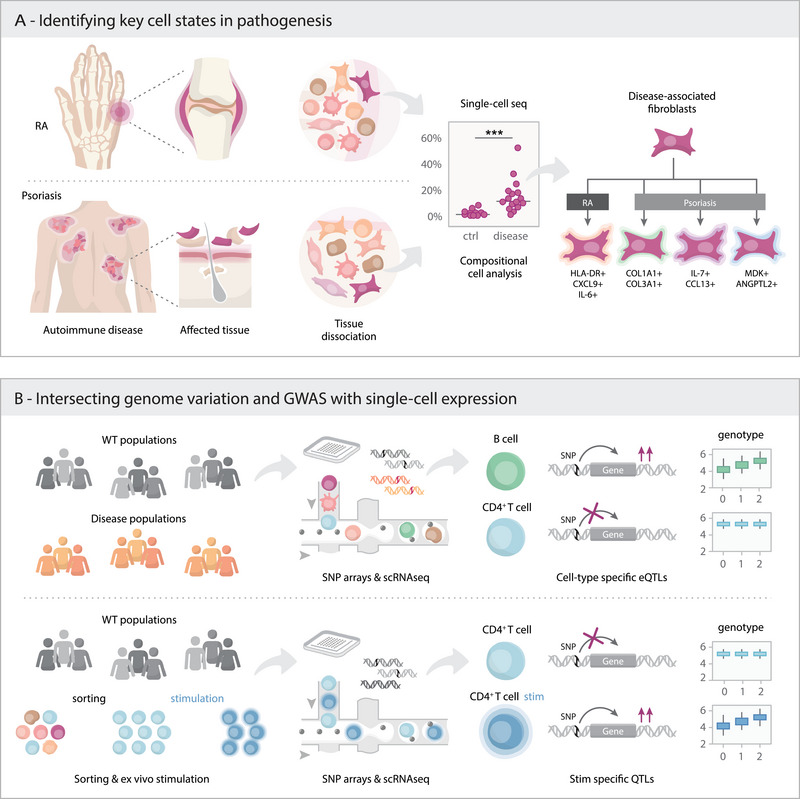
Cell states and genetic intersections in autoimmunity through single‐cell technologies. (A) Workflow for the isolation and characterization of the cellular composition of tissue biopsies using single‐cell sequencing. Briefly, cells are obtained by dissociation of a biopsy of healthy or disease‐affected tissue. Downstream compositional analysis can reveal populations of cells significantly enriched in the disease group. Disease‐associated fibroblasts reported in rheumatoid arthritis (RA) and psoriasis using single‐cell approaches are highlighted as an example. (B) Profiling of genetically diverse populations of healthy and disease‐affected individuals with single‐cell sequencing enables the discovery of genetic variants (shown as single‐nucleotide polymorphisms, SNP) that regulate gene expression in either a cell type‐specific manner or a cell state‐specific manner (bottom panel). These gene regulation effects are known as cell type‐specific or cell state‐specific expression quantitative trait loci (eQTL). Examples show a B‐cell type‐specific eQTL (top panel) and a stimulation‐specific eQTL in CD4+ T cells (bottom panel). The co‐localization of these regulatory variants with variants associated with disease by genome‐wide association studies (GWAS) provides insights into the genetic basis of disease.

The application of single‐cell genomics to autoimmunity patients is shedding light on disease pathogenesis. These techniques allow the dissection of heterogeneous cell populations and the identification of cell states and gene expression programs enriched in diseased tissues (Figure 1A). The studies we discuss below have not only added depth of molecular information in well‐known autoimmunity‐related cell types, such as antigen‐specific lymphocytes but have also expanded our understanding of the contribution of stromal cells and their interactions with immune cells.

### 2.1 Stromal Cell States

A prime example of these advancements has been the discovery of specific subtypes of fibroblasts that beyond their structural role [7] contribute to inflammation [8, 9, 10, 11] and antigen presentation [12, 13]. Recent scRNAseq studies have revealed the expansion of HLA‐DR^high^ fibroblasts in synovial tissue of rheumatoid arthritis (RA) patients with active disease [9, 14–16]. Multimodal analysis of this population by scRNAseq and single‐cell assay for transposase‐accessible chromatin sequencing (scATAC‐seq) revealed enrichment of IFN signalling and increased chromatin accessibility for downstream transcription factors such as STAT1, FOS and JUN [17]. Furthermore, these fibroblasts support T‐ and B‐cell recruitment and survival through the secretion of chemokines (CXCL9 and CXCL12) and cytokines (IL‐6, IL‐15), while receiving inflammatory signals that reinforce their pathogenic phenotype [16, 17]. In some of these RA studies, the dissection of immune‐stromal interactions has also been addressed using spatial transcriptomics methods, adding information on the tissue context where these cells are found and into neighbouring cells with which they may interact [16]. Spatial studies have also offered insights into the cellular landscape of synovial tertiary lymphoid structures (TLSs) [15], which are organized ectopic lymphoid aggregates arising at sites of chronic inflammation and autoimmune lesions [18].

Several fibroblast subtypes have also been defined in the context of psoriasis. In a study combining scRNAseq with spatial transcriptomics of healthy and psoriasis skin biopsies, the authors identified two subtypes of SFRP2^+^ fibroblasts enriched in lesional skin: one expressing collagen genes (*COL1A1, COL3A1*) and another secreting chemokines (*CCL13, CXCL12*) and the cytokine IL‐15. The latter localized in the upper dermis, where they may be involved in recruiting T cells and myeloid cells [19]. A smaller psoriasis study suggests an additional role of fibroblasts in supporting angiogenesis in the lesions given their production of angiopoietins (MDK, ANGPTL2), whose receptors were expressed in endothelial cells and keratinocytes [20]. Some of the fibroblast phenotypes reported above have also been observed in additional studies of atopic dermatitis (AD), such as COL1A1^+^ fibroblasts [21], or in the related conditions localized scleroderma (LS) and systemic sclerosis (SSc) where SFRP2^+^ and CCL19^+^ fibroblasts have been previously found [22, 23].

Given the recurrent phenotypic features found across autoimmune conditions and the relatively small size of most single‐cell disease studies, an integrative approach that can map the spectrum of cell states across studies and diseases could provide critical insights into their roles in inflammation. A first attempt in this direction is represented by a cross‐tissue atlas of pathological fibroblasts in four chronic inflammatory diseases, including RA, Sjögren's syndrome, inflammatory bowel disease and interstitial lung disease [24]. Fibroblast subtypes identified across the four tissues (synovium, salivary glands, intestine and lung) associated with each disease, respectively, included pro‐inflammatory (CXCL10^+^CCL19^+^) and vascular‐interacting phenotypes (SPARC^+^COL3A1^+^).

### 2.2 Immune Cell States

We have just discussed how some of the emergent roles of stromal cells in autoimmunity relate to their capacity to produce paracrine signals for recruitment (e.g., CCL19:CCR7 axis, CCL13:CCR5 axis) and survival (e.g., IL‐15) of immune cells. Next, we will discuss evidence involving specific immune cell states in both affected tissues and blood of autoimmunity patients.

The presence of autoantibodies is a hallmark of autoimmunity [25, 26, 27, 28, 29], however, the identity of disease‐associated B cells responsible for autoantibody generation remains elusive. CD11c^+^T‐bet^+^ B cells, also known as atypical, autoimmunity‐associated B cells or age‐associated B cells (ABCs) [9], are a unique subset of double negative memory B cells (IgD^−^CD27^−^) increasingly recognized as crucial mediators of autoimmunity. ABCs are enriched in systemic lupus erythematosus (SLE) [30] and RA [9, 14, 31]. In SLE, ABCs are thought to play a central role in extrafollicular immune responses and together with other cell types, such as plasma cells, exhibit an interferon‐stimulated genes (ISGs) expression signature that correlates with disease activity in adult and pediatric cohorts [30]. In RA, ABCs have been observed in synovial biopsies of chronic patients [9, 14]; however, in synovial samples obtained at diagnosis, double negative memory B cells do not show the ABC phenotype, suggesting that this cell state may be reflective of chronic inflammation or therapeutic influence [14, 32]. Apart from the recently appreciated role of ABCs, memory B cells and plasma cells are also found in autoimmune lesions, in particular, in TLSs [15]. Nonetheless, the clonal relationships between these different B‐cell states are poorly understood. Single‐cell sequencing allows querying of clonality, thanks to the concomitant detection of antigen receptors and transcriptomic profiles. In a recent RA study combining scRNAseq and scVDJseq, the largest clonal expansions in synovial lymphocytes were found in plasmablasts and plasma cells, however, clonal sharing between memory B cells, activated B cells and ABCs was also detected [31]. This work showcases how deep and unbiased repertoire and phenotypic analysis of lymphocytes could help disentangle how autoantigen‐specific responses develop in affected tissues.

T cells constitute another fundamental pillar determining the development of autoimmunity, with CD4^+^ T cells supporting B‐cell activation and both CD4^+^ and CD8^+^ T cells undertaking cytotoxic responses in affected tissues. Together with PD1^hi^CXCR5^+^CXCL13^+^ follicular helper T (T_fh_) cells, PD1^hi^CXCR5^−^CXCL13^+^ peripheral helper T (T_ph_) cells have emerged as a distinct pathogenic CD4^+^ T cell population with the ability to drive plasma cell differentiation. Initially described by a combination of mass cytometry and transcriptome profiling in the synovium of RA patients [33], T_ph_ are thought to attract follicular‐like CXCR5^+^ B cells into the joint via the CXCL13:CXCR5 axis. Reassuringly, the frequency of T_fh_/T_ph_ correlates with ABC cell counts in RA synovium [14]. Remarkably, scRNAseq has found further heterogeneity within T_ph_ cells in the synovial fluid of RA patients, with levels of GPR56 delineating diverse T_ph_ cell states [34]. In the same study, the authors assessed the clonality of the CD4^+^ T‐cell compartment and found clonal sharing between T_ph_ cell states and to a lesser extent between T_ph_ and cytotoxic CD4^+^ T cells. Another RA study found that clonally expanded T_ph_ cells show higher cytotoxicity gene signatures than their unexpanded T_ph_ counterparts [31]. In addition, cytotoxic CD4^+^ T cells have been found in other studies analysing synovium and peripheral blood of RA patients [14, 34, 35] as well as cerebrospinal fluid (CSF) and peripheral blood of relapsing‐remitting multiple sclerosis (MS) patients [36, 37]. Clonal expansions detected in this population also point to a key role in driving pathogenesis [31, 34, 37].

Along the lines of the recurring cellular phenotypes across diseases discussed above for fibroblasts, the observations regarding the CD4^+^ T cells have motivated a meta‐analysis of CD4^+^ T cells across immune‐mediated conditions. The study integrated peripheral CD4^+^ T cells from healthy individuals, autoimmunity patients (MS, SLE and myasthenia gravis) and other conditions [38] and created a machine learning model to predict T cell identity, representing a promising step towards integrating results across studies. These findings underscore the shared phenotype of cell states across different diseases, paving the way for the development of therapies targeting a common pathogenic phenotype, with the potential to treat multiple immune‐mediated conditions.

Our understanding of the involvement of CD8^+^ T cells in autoimmunity has also been boosted by scRNAseq studies. Notably, those combining simultaneous transcriptomic and repertoire profiling. In the synovium of RA [31] and psoriatic arthritis [39] patients and in the CSF of MS patients, CD8^+^ memory T cells show larger clonal expansions in comparison to CD4^+^ T cells [37]. Furthermore, these expanded clones exhibit signatures of cytotoxicity and effector function compared with their unexpanded counterparts [31, 37]. Related activation gene signatures have been found as well in expanded synovial T cells of juvenile idiopathic arthritis (JIA) patients [40]. Together with the transcriptomic and clonality studies in B cells and CD4^+^ T cells, these studies contribute to building an increasingly accurate picture of the dynamics of antigen‐specific lymphocyte activation, expansion and tissue adaptation in autoimmune conditions.

In addition to defining major cell subsets with an increasing level of detail, single‐cell sequencing also enables the dissection of rare cell populations. This is the case of specific subsets of dendritic cells (DCs) that are essential for the activation of autoreactive T cells. In lesional skin from AD and psoriasis patients, scRNAseq revealed increased frequencies of LAMP3^+^BIRC3^+^ immunoregulatory DCs, representing a major source of IL15, and CD14^+^ DC3 cells, expressing the cytokines IL1β and IL23A [41]. In another AD examining skin from patients receiving dupilumab treatment (IL4RA blocking antibody), scRNAseq revealed that LAMP3^+^IL15^+^ DCs remained in the tissue alongside T cells after treatment, suggesting that these populations may contribute to disease recurrence [42]. LAMP3^+^ DCs have also been reported in blood and CSF samples of inflammatory demyelinating disease (IDD) patients, including MS [43]. Nonetheless, in these patients, this DC subset is not increased compared with controls. Instead, it is a population of AXL^+^SIGLEC6^+^ DCs (ASDCs) that is enriched in IDD.

In conclusion, single‐cell sequencing has significantly expanded our cellular phenotyping ability for autoimmune diseases by identifying specific immune cell states and molecular mechanisms involved in disease pathogenesis. In the future, these findings together with further analysis and integrative approaches have the potential to inform the development of targeted therapies and the selection of biomarkers for monitoring disease progression and treatment.

## 3 Intersecting Cell State Phenotypic Data with Genetic Associations

Genome‐wide association studies (GWAS) have yielded a wealth of genetic associations with autoimmune conditions and integration of these with functional genomic datasets has pointed to specific immune cell subsets as the context in which these genetic associations manifest functionally [44] This information has proven highly advantageous in the prioritization of drug targets given the higher success rate of targets supported by genetics data [45]. An approach to decode the mechanisms mediating those associations has been to intersect GWAS signals with functional genomics readouts. Given that some of the gene programs affected are highly cell‐type and cell‐state‐specific, it is thought that applying single‐cell genomics to relevant biological specimens may unlock the molecular details underpinning these. More recently, the intersection of this with the cell type specificity of target gene expression has been proposed as an additional avenue to increase the clinical success of new [46]. In the previous section, we have discussed small‐scale cohort studies that have been fundamental to capturing disease‐related cell states in relevant tissues. In this section, we move towards emerging larger‐scale cohort studies connecting human genetics and high‐resolution phenotyping that promise to cause a paradigm shift for therapeutics of autoimmunity [47]^.^ In this research direction, there are different study designs with regard to the subjects being recruited (healthy individuals or patients) as well as in relation to the type of ex vivo profiling (steady state or upon stimulation). The common strategy is that the single‐cell profiles are then intersected with the genetic variation of the profiled individuals to detect quantitative trait loci (QTL) that are then intersected with GWAS association signals (Figure 1B).

In this space, there are steady‐state profiling or perturbation studies. An elegant example of the first type of study focused on memory T cells in a cohort of 259 individuals [48]. The most remarkable finding of this study is that T cells found in a diverse spectrum of activation states show different enrichments for GWAS signals, suggesting that the gene expression programmes involved in disease pathogenesis are only active in a dynamic manner. Another key study pointing to the importance of calculating QTLs across dynamic processes analysed naive and memory CD4^+^ T cells from 119 individuals at three different stimulation timepoints by scRNAseq. Results revealed genetic variants that modulate gene expression (expression quantitative trait loci (eQTLs)) and perform colocalization of these signals with previously generated GWAS of immune‐mediated conditions [49]. Another interesting perturbation study generated 1.3 M PBMCs from 120 individuals longitudinally exposed to *Candida albicans*, *Mycobacterium tuberculosis*, and *Pseudomonas aeruginosa*. In monocytes, where there was a good representation of cells per donor (>100), the authors could identify not only QTLs specific to the pathogen response context but also co‐expression QTLs. Specifically, this study revealed a co‐expression QTL for *CLEC12A*, expressed by monocytes and dendritic cells, with several IRF genes and correlation with polygenic risk score of SLE, potentially linking exposure to these pathogens with autoimmunity [50].

Steady‐state profiling at an unprecedented scale was achieved by the Onek1k study including over 1 million PBMCs from 982 individuals [51]. This work revealed genetic variants controlling gene expression in a cell type‐specific manner and the colocalization of these signals with autoimmunity GWAS, such as *ORMDL3* eQTLs in B cells and CD8^+^ T cells. Another remarkable study performed on steady‐state PBMCs but included in this case patient samples analysed a cohort comprising 162 SLE cases and 99 healthy controls [52]. This study not only allowed for patient stratification but also revealed unique cell type‐specific eQTL signals. On a much smaller scale, single‐cell genomics has also started to be applied to monogenic autoimmune conditions such as Helios deficiency, manifesting as an SLE‐like condition [53].

A different strategy was followed in an extensive cross‐tissue analysis of single‐cell profiles in 16 individuals where the authors calculated cell type enrichment for genes linked to pre‐existing QTL data overlapping disease‐associated GWAS loci, including autoimmune conditions such as diabetes type 2 and psoriasis [54]. This study is the first of its kind and gives a complete perspective of cell type specificity considering a broad range of tissues and cell types under the same experimental setting, potentially increasing the power to identify exquisitely specific drug targets. Another frontier in single‐cell studies considering genetic variation is the dissection of effects on cellular interactions. The identification of this type of multicellular network is the goal of a tool called DIALOGUE which was applied to ulcerative colitis (UC) and linked to genetic risk loci for developing the disease and for specific disease trajectories relevant to prognosis [55]. Overall, these studies open the door to unlocking the coordination of cell type‐specific and intercellular communication networks anchored on population genetics data that may become robust targets in a therapeutic context.

## 4 Antigen‐Specificity in the Context of Single‐Cell Genomics

Clinical diagnosis and monitoring of autoimmune conditions are often based on the detection of autoantibodies [29]. Furthermore, most autoimmune diseases are linked to HLA risk alleles [56] and clonal expansion of T and B cells in affected tissues. All these together highlight the key contribution of adaptive immune cells to these conditions. Nonetheless, we still have a limited understanding of the cell states that initiate these responses. Our ability to analyze T‐ and B‐cell receptor repertoires has greatly advanced in the last decade. Initially, bulk approaches focused on sequencing the CDR3 region of TCR‐b or the heavy immunoglobulin chain [56, 57], providing insights into the diversity of the TCRs/BCRs and clonal architecture of autoreactive lymphocytes. More recently, single‐cell approaches have enabled the detection of paired chains while also offering the opportunity to evaluate transcriptomic phenotype and antigen specificity at the individual cell level. We discuss here the specific advantages of single‐cell profiling that are relevant to decoding mechanisms underpinning autoimmunity.

**FIGURE 2:**
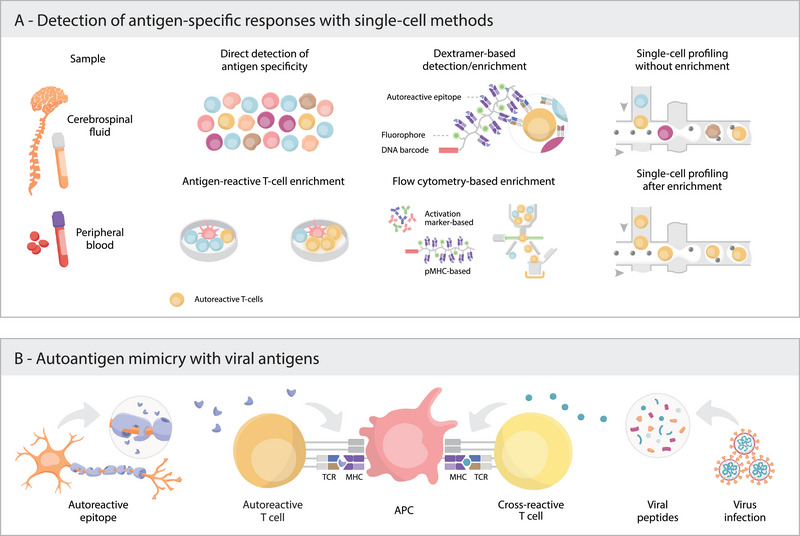
Antigen‐specificity in autoimmunity. (A) Schematic of different approaches used for profiling autoreactive T cells from tissue biopsies (cerebrospinal fluid and peripheral blood are shown as examples). Samples can be analysed directly ex vivo using without enrichment (“Direct detection of antigen specificity”) or after ex vivo exposure to autoantigens to activate and/or expand antigen‐specific lymphocytes (“antigen‐reactive T‐cell enrichment”). Direct detection methods require the use of peptide‐MHC (pMHC) tetramers or dextramers marked with a DNA barcode compatible with single‐cell sequencing and flow cytometry. Flow cytometry‐based sorting offers the possibility to enrich cells based on activation marker expression or pMHC binding. Downstream single‐cell sequencing can be applied on samples with or without prior enrichment and it enables the simultaneous characterization of transcriptomes, antigen receptor sequence and antigen specificity. (B) Activation of autoreactive T cells following infection can occur due to cross‐reactivity between self‐antigens and pathogen‐derived antigens, a mechanism known as molecular mimicry. An example is the cross‐reactivity between myelin antigens and viral peptides, which can trigger autoimmune responses. APC, antigen‐presenting cell.

Classically, antigen‐specific T cells have been identified with MHC tetramers [58, 59, 60], allowing the sorting and bulk analysis of these cells. This has most successfully been achieved in the context of viral infections, where antigenic peptides are well characterized [61, 62]. Conversely, in autoimmune diseases, very few self‐peptides are known [63] making it difficult to effectively hone into these cells. In addition, autoantigen‐specific T cells are typically present at low frequency, requiring ex vivo expansion or activation prior to profiling (Figure 2A). This can result in changes to the cellular phenotype of the antigen‐specific T cells, leading to the recent development of protocols to minimize the ex vivo manipulation of these cells [64, 65, 66].

Computational tools provide a complementary approach to gain insight into TCR sequences by specificity and define disease‐associated repertoires [67]. For instance, GLIPH and TCRdist algorithms have been developed to predict clusters of TCRs with structural similarity and shared specificity across different antigens, enabling in silico identification of targets [68, 69]. However, both tools rely on data containing TCR sequences linked to viral antigens, potentially limiting the prediction power for specificity against self‐peptides, which were not encountered during the training phase. In general, antigen‐specificity databases still offer a limited representation of the full range of pathogenic epitopes [70, 71]. Recently, significant efforts based on immunopeptidomics have led to the discovery of a panoply of new antigens, including autoantigens, promising to expand our understanding of epitope‐to‐antigen receptor interactions [63].

Single‐cell sequencing‐compatible readouts enable precise identification of antigen specificity at the single‐cell level, overcoming some limitations of traditional bulk approaches. Some studies have combined antigen‐reactive T‐cell enrichment (by ex vivo exposure of T cells to the autoantigen) with single‐cell sequencing, enabling an unbiased identification of autoreactive T‐cell specificity without the need to use tetramer‐based enrichment. For instance, a recent study on neuromyelitis optica spectrum disorder (NMOSD) successfully characterized aquaporin 4 (AQP4)‐reactive T cells and found evidence of oligoclonal T cell responses associated with an exhaustion gene signature [72]. Similarly, another study on autoimmune hepatitis (AIH) identified T cells activated upon ex vivo exposure to epitopes derived from the soluble liver antigen (SLA), which consisted of multiple clonotypes across different patients exhibiting a T_ph_ phenotype [73]. Along the same lines, a study on type 1 diabetes (T1D) used an ex vivo activation approach with the known diabetes antigen glutamic acid decarboxylase 65 (GAD65) and incorporated specificity prediction with the GLIPH algorithm. The authors identified GAD65‐reactive T cells at similar frequencies in T1D patients and healthy individuals [74]. GLIPH analysis revealed clusters of TCRβ CDR3 regions targeting the same epitopes and were found in naive as well as memory T‐cell populations, suggesting a physiological role for this GAD65‐reactive T cells that may become dysregulated upon additional triggers in T1D [74].

Following a similar approach but in this case with longer expansion in vitro, memory CD4^+^ T cells from Guillain–Barré syndrome (GBS) patients were assessed for reactivity against peripheral nervous system (PNS)‐myelin peptide pools [75]. scRNAseq revealed that GBS patients have PNS‐myelin‐reactive CD4^+^ T cells in the blood and CSF showing a signature of cytotoxic T helper type 1 (T_h1_)‐like cells. Further analysis of TCRβ repertoire in isolated T cell clones and PBMCs from GBS patients detected cross‐reactivity of PNS‐myelin‐reactive T cells with CMV epitopes, also supported by TCR specificity groups found by the GLIPH2 algorithm [76]. By linking these clonotypes to both viral and self‐antigens, this work supports a connection between infections and autoimmunity [77, 78, 79]. Molecular mimicry occurs when autoreactive lymphocytes recognize foreign antigens and self‐peptides (Figure 2B). This mechanism has been associated with different autoimmune diseases, where infections may act as triggers by breaking immune tolerance and promoting aberrant immune responses against self‐tissues [80, 81, 82, 83, 84].

The assessment of antigen specificity coupled with scRNAseq without prior in vitro exposure to antigens can be achieved with DNA‐barcoded MHC multimers (tetramers or dextramers) [85] (Figure 2A). These potentially allow for the screening of a large number of epitopes in the same sample and link antigen specificity with the transcriptomic profile of antigen‐specific T cells. A key study in this area showcased a tetramer‐based screening of 280 antigens combined with scRNAseq, which found T1D autoantigen‐specific CD8^+^ T cells with similar phenotypes between T1D patients and healthy individuals [86]. Interestingly, the authors also found evidence of cross‐reactivity between diabetes‐related antigens and peptides derived from the gut microbiome bacteria *Bacteroides fragilis* and *Bacteroides thetaiotaomicron*.

We have not yet achieved the ability to map B‐cell antigen specificity to the same extent as T‐cell antigen specificity. This is related to the unique challenges of modelling antigen‐specific B cell responses due to antibody epitopes potentially involving any surface‐exposed region of an antigen, in any of its conformations [87]. Nonetheless, recent advances in paired‐chain BCR sequencing and proteomics have led to the identification of cross‐reactive antibodies. Specifically, a monoclonal antibody in the CSF of an MS patient was found to bind the EBNA1 epitope of Epstein Barr virus (EBV) and cross‐react with GlialCAM (protein glial cell adhesion molecule), confirming that 20–25% of MS patients possess cross‐reactive antibodies against EBV and GlialCAM [88]. This finding strongly supports the role of molecular mimicry in the pathogenesis of MS [82, 83].

Altogether, the studies above highlight how the precise analysis of antigen‐specific responses at an individual cell level can uncover mechanisms underpinning autoimmunity, including molecular mimicry, enhancing our understanding of disease pathways and opening avenues for targeted therapies by identifying specific autoreactive cell subsets and their interactions with environmental triggers.

## 5 Conclusion

In this review, we have presented three strategic domains of knowledge fundamental for dissecting autoimmune pathology where single‐cell technologies have had an impact in recent years: (1) the unprecedented level of detail in the molecular phenotyping of cells in patient tissues; (2) the novel strategies to link genetic association data from population studies with cellular phenotypes; and (3) the integrative analysis of antigen specificity data with the high‐resolution of single‐cell sequencing methods.

Two aspects that remain untapped territory are patient heterogeneity and changes along disease trajectories. We anticipate that with the expansion of single‐cell approaches to larger cohorts, including longitudinal, the phenotypic variability will be tackled in a more systematic way. As a complement to the analysis of bigger cohorts, we strongly believe that computational integration across multiple studies, such as those highlighted above [24, 38], will become commonplace. These efforts will not only generate datasets of increasing size but also more robust datasets from which advanced deep‐learning tools can extract informative patterns for disease stratification and drug prioritization [89].

In the area of genetic studies, the dissection of loci controlling gene expression at single‐cell resolution is reaching new heights with 2000 individuals included in the latest study, expanding the number of cell type‐specific eQTLs that can be used for the interpretation of disease‐associated variants [90]. Nonetheless, an important limitation of this type of study is that while they are performed in blood cells, many of the genetic mechanisms acting in autoimmune pathology may be active only in specific tissue contexts. We foresee two complementary future directions to overcome this limitation by accounting for tissue‐specific genetic mechanisms. On the one hand, the establishment and scaling of tissue biobanks for both healthy and disease samples stemmed from the highly successful work of the GTEX consortium [91]. On the other hand, the development of robust and scalable disease models can replace or, at least, complement primary tissue studies, as recently proposed by researchers working at the intersection of stem cell biology and genetics [92].

## Author Contributions

All authors have read and agreed to the published version of the manuscript.

## Conflicts of Interest

The authors declare no conflicts of interest.

## Peer Review

The peer review history for this article is available at https://publons.com/publon/10.1002/eji.202451233.

## Data Availability

Data sharing is not applicable to this article as no datasets were generated or analysed during the current study.
